# Chitosan Treatment Promotes Wound Healing of Apple by Eliciting Phenylpropanoid Pathway and Enzymatic Browning of Wounds

**DOI:** 10.3389/fmicb.2022.828914

**Published:** 2022-03-02

**Authors:** Sabina Ackah, Sulin Xue, Richard Osei, Francis Kweku-Amagloh, Yuanyuan Zong, Dov Prusky, Yang Bi

**Affiliations:** ^1^College of Food Science and Engineering, Gansu Agricultural University, Lanzhou, China; ^2^College of Plant Protection, Gansu Agricultural University, Lanzhou, China; ^3^Department of Food Science and Technology, University for Development Studies, Tamale, Ghana; ^4^Department of Postharvest Science of Fresh Produce, Agricultural Research Organization, Rishon LeZion, Israel

**Keywords:** chitosan, apple fruit, wound healing, phenylpropanoid metabolism, enzymatic browning

## Abstract

Chitosan is an elicitor that induces resistance in fruits against postharvest diseases, but there is little knowledge about the wound healing ability of chitosan on apple fruits. Our study aimed at revealing the effect of chitosan on the phenylpropanoid pathway by determining some enzyme activities, products metabolites, polyphenol oxidase activity, color (L*, b*, a*), weight loss, and disease index during healing. Apple (cv. Fuji) fruits wounded artificially were treated with 2.5% chitosan and healed at 21–25°C, relative humidity = 81–85% for 7 days, and non-wounded fruits (coated and non-coated) were used as control. The result shows that chitosan treatment significantly decreased weight loss of wounded fruits and disease index of *Penicillium expansum* inoculated fruits. The activities of phenylalanine ammonia-lyase (PAL), cinnamic acid 4-hydroxylase (C4H), 4-coumaryl coenzyme A ligase (4CL), cinnamoyl-CoA reductase (CCR), and cinnamyl alcohol dehydrogenase (CAD) were elicited throughout the healing period by chitosan, which increased the biosynthesis of cinnamic acid, caffeic acid, ferulic acid, sinapic acid, *p*-coumaric acid, *p*-coumaryl alcohol, coniferyl alcohol, and sinapyl alcohol. Also, total phenol, flavonoid, and lignin contents were significantly increased at the fruits wounds. In addition, chitosan’s ability to enhance polyphenol oxidase activity stimulated enzymatic browning of wounds. Although wounding increased phenylpropanoid enzymes activities before healing, chitosan caused higher enzyme activities for a significant healing effect compared with the control. These findings imply that chitosan accelerates apple wound healing by activating the phenylpropanoid pathway and stimulating enzymatic browning of wounds.

## Introduction

Apple (*Malus domestica* Borkh.) is the most economically widely produced fruit crop in temperate zones (Wang et al., 2018). However, apples are susceptible to mechanical injury during harvest and postharvest handling. Wounds formed on the fruit’s surface create a channel for the invasion of pathogens that causes rot, which further results in excessive water evaporation (Gong et al., 2019). Apples have the ability of wound healing by accumulating lignin and polyphenols to prevent the invasion of fungi (Zhang et al., 2020). However, the natural wound healing process takes a long time; therefore, rapid wound healing is critical for apple postharvest quality.

Chitosan is a chitin N-deacetylated derivative obtained from crustaceans’ outer shells, mainly shrimps, crabs, krills, some fungi cell walls, etc. It is a natural polymer with a polycationic nature, non-toxic, biodegradable, excellent film-forming property, and antimicrobial effect (Deepmala et al., 2014; Silva and Pighinelli, 2017). Chitosan is used as an elicitor to induce resistance against postharvest diseases of horticultural crops (Romanazzi et al., 2017), such as gray mold caused by *Botrytis cinerea* in tomatoes, grapes, strawberries, apples, and peaches (Plainsirichai et al., 2014; Zhang et al., 2015; Gayed et al., 2017; Peian et al., 2021). Similarly, chitosan induced resistance to anthracnose caused by *Colletotrichum gloeosporioides* in citrus (Zhao et al., 2018), blue mold caused by *Penicillium expansum* in apples (Lu et al., 2014; Li et al., 2015), and green and blue mold caused by *Penicillium digitatum* and *Penicillium italicum* in orange (Zeng et al., 2010). Chitosan acts directly on the pathogens as a fungicide to destroy fungus (Li et al., 2015). Also, chitosan induces signal transduction pathways such as jasmonic acid (JA), salicylic acid (SA), ethylene (ET), and hydrogen peroxide to activate systemic acquired resistance of fruit in grapes and strawberries in response to stress (Gu et al., 2021; Peian et al., 2021). Moreover, chitosan residues bind to fruits cell membranes to stimulate the synthesis of phytoalexin and pathogenesis-related proteins: chitinase, β-1,3-glucanase, and lignin biosynthesis and activates reactive oxygen species generation in fruits (Li et al., 2015). Enzymatic browning, which results from the oxidation of phenolic compounds to quinones, is catalyzed by polyphenol oxidase (PPO) with brown pigmentation (Yan et al., 2018). Chitosan increases not only the synthesis of phenolic compounds but also PPO activity to stimulate browning in apples and citrus (Pilon et al., 2015; Zhao et al., 2018). Previous studies showed that chitosan elicited phenylalanine ammonia-lyase (PAL) activity to increase the content of phenolic compounds in tomatoes, grapes, and strawberries (Coqueiro et al., 2015; Romanazzi et al., 2017). Chitosan’s efficacy in treating fruits has been demonstrated in numerous studies and has proven that chitosan coating can prevent deterioration and extend the shelf life of a range of fruits and vegetables (Plainsirichai et al., 2014; Li et al., 2015; Zhang et al., 2015; Gu et al., 2021). Moreover, the recent work by our team reveals that preharvest spraying with chitosan accelerated the wound healing process of muskmelons after harvesting by eliciting phenylpropanoid metabolism for the production of phenolic compounds and lignin at the wounded site (Li et al., 2021). Although chitosan antifungal effect has been demonstrated in many whole fruits and preharvest spraying with chitosan has been reported to promote wound healing in harvested muskmelons, there is no report on chitosan wound healing ability on apple fruits. Thus, this study aimed at exploring chitosan’s effects on the wound healing of apple fruits. Fruits were artificially wounded, treated with 2.5% chitosan, and stored at room temperature of 20 ± 5°C, relative humidity (RH) = 81–85% to heal. The (1) weight loss of wounded/non-wounded fruits and disease index of *P. expansum* inoculated fruits were evaluated, (2) some important enzyme activities and products metabolites were determined, and (3) PPO activity and color (L*, b*, a*) of wounds during healing were subsequently analyzed.

## Materials and Methods

### Fruit and Chitosan

Apple (*M. domestica* Borkh. cv. Fuji) fruits were harvested from a commercial orchard of Tiaoshan farm in Jingtai (37°380 N, 105°340 E, 1,671 m altitude), Gansu Province, China. Fruits that are free from disease and injury, uniform size, and maturity were sorted, and each fruit was netted with foam. The fruits were immediately packed in perforated paper boxes with each box containing 36 fruits, transported to Gansu Agricultural University, and stored at 2 ± 4°C, RH = 80–85%.

Water-soluble chitosan (degree of deacetylation ≥90%, WN Group of Publishers Ltd., Mansouriah, France) was used as treatment. Twenty-five gram of chitosan was completely dissolved in 1,000-ml H_2_O to make a chitosan solution. The concentration of 2.5% chitosan used was based on preliminary test results.

### Wounding and Treatment of Fruits

Wounding and treatment of apples were done according to Zhang et al. (2020). The fruits were first washed with water, disinfected using 0.1% (v/v) NaClO_2_ for 3 min, then rinsed with water and allowed to dry. After drying, each fruit was disinfected with 75% ethanol. A sterilized scalpel (Deli, no. 2034, China) was used to make three wounds on the equatorial region of each fruit (circle 7.3–7.8-mm radius and 1-mm depth) and then soaked in 2.5% (w/v) chitosan for 10 min and allowed to dry on a clean surface. Two independent experiments were conducted in a completely randomized design with three replicates. The treatments were (i) wounded then treated with chitosan (W + chitosan), (ii) wounded then treated with distilled water (W + water), (iii) non-wounded and treated with chitosan (N + chitosan), and (iv) non-wounded and treated with distilled water (N + water). All fruits were kept in aerated poly-ET bags and subsequently stored at room temperature at 20–25°C, RH = 81–85% to heal.

### Determination of the Rate of Water Loss

The determination of the weight loss of wounded and non-wounded fruits was done by weighing fruits gravimetrically according to Zhang et al. (2020) using an electronic scale (HZ electronic LLC, ST Hartford, CT, United States) on 0, 1, 3, 5, and 7 days of healing. The fresh weight change of apple fruits at each time point was divided by the initial weight and expressed as a percentage and recorded as the total weight loss. Ten fruits from each treatment were weighed at each time point and were repeated three times.

### Determination of Diseases Index

The method reported by Zhang et al. (2020) for disease index was used. A spore suspension (1 × 10^5^ spores/ml) of *Penicillium expansum* was prepared. Then, the wound surfaces of fruits were inoculated with 20-μl spore suspension on 0, 1, 3, 5, and 7 days of healing. The inoculated fruits were then packed in aerated poly-ET bags and kept at 21–25°C, RH = 81–85% in the dark for incubation. Infected fruits were physically observed after 5 days of inoculation. The incidence was categorized in five levels based on the extent of fungi growth, that is: grade 4, fungi on all the wound surface; grade 3, fungi on three-fourths of wound surface; grade 2, fungi on a one-half area of wound surface; grade 1, fungi growth on a one-fourth area of wound surface; and grade 0, no fungi growth on the wound surface. Disease index was calculated using the formula, disease index = [Σ (the number of wounds at the disease level × the level of disease)]/4 × total wounds] × 100%. Ten wounds were evaluated at each time point and were repeated three times.

### Determination of Wound Surface Color

The wound surface color was determined according to Dea et al. (2010) with slight modification. A colorimeter (X-Rite Ci6X, United States) was used to determine the color of the individual wounds of fruits on 0, 1, 3, 5, and 7 days of healing. L* (lightness), a* (reddish–greenish), and b* (yellowish–bluish), Chroma C* = [(a)2 + (b)2]1/2 and hue angle *h*°= [tan – 1(b*/a*)] data were collected to evaluate the wound surface color. Fifteen wounds were observed at each time point and were repeated three times.

### Sampling

Fruits wound tissues were collected according to Zhang et al. (2020) after 0, 1, 3, 5, and 7 days of healing by carefully separating the scarred outmost tissue from the cut surface using a sterilized sharp blade. Tissues of non-wounded fruits were collected. Nitrogen (liquid) was used to freeze the collected tissues, ground with a grinder (IKA, no. A11, Germany) into a powdery form, and subsequently stored at −80°C until use.

### Hydrogen Peroxide Content Assay

Hydrogen peroxide (H_2_O_2_) content was determined using a test kit from Suzhou Keming Biotechnology Co., Ltd., Suzhou, China. Extract solution (1 ml) was added to 0.1 g of frozen sample, then centrifuged at 8,000 × *g* at 4°C for 10 min. The supernatant was used as a crude enzyme. Other reagents were added as instructed. The result was expressed as μmol/g FW.

### Assay of Some Enzymatic Activities

Phenylalanine ammonia-lyase activity was determined using the Koukol and Conn’s (1961) method. Three milliliters of 100 mmol/L borate buffer (pH 8.8, 40 g/L polyvinylpyrrolidone, 2 mmol/L ethylenediaminetetraacetic acid, and 5 mmol/L β-mercaptoethanol) was added to 1-g frozen powder for 2 h to allow extraction, then centrifuged at 4°C at 8,000 × *g* for 30 min. The supernatant was used as a crude enzyme. PAL was assayed by adding 3 ml of 50 mmol/L borate buffer (pH 8.8) and 0.5 ml of 20 mmol/L L-phenylalanine to 0.5 ml of crude enzyme. Absorbance, optical density at a wavelength of 280 nm (OD_290_) was measured immediately, and just after keeping in a 37°C water bath for 1 h, the second OD_290_ was measured. PAL activity was expressed as U/g FW, where *U* = 0.01 OD_290_ min^–1^.

Cinnamic acid 4-hydroxylase (C4H) activity was determined using a kit from Beijing Solarbio Science & Technology Co., Ltd., China. Extract solution (1 ml) was added to 0.5 g of frozen powder, centrifuged at 11,000 × *g* at 4°C for 15 min, and the collected supernatant was the enzyme solution. Regents were added as instructed. C4H activity was expressed as U/g FW, where *U* = 0.01 OD_340_ min^–1^.

4-Coumaryl coenzyme A ligase (4CL) activity was assayed using the method of Liu et al. (2014) with modifications. Three milliliters of extraction solution (Shanghai Yuanye Bio-Technology Co., Ltd., Shanghai, China) was added to 1 g of a frozen powder for 1 h and centrifuged at 8,000 × *g* for 30 min at 4°C, and the resulting supernatant was collected as the enzyme solution. An amount of 0.15 ml of 5-mM *p*-coumaric acid, 0.15 ml of 1-mM CoA, 0.15 ml of 50-mM adenosine triphosphate, and 0.45 ml of 15-mM MgCl_2_•6H_2_O was added to a 0.5-ml crude enzyme solution, kept for 30 min at 40°C. 4CL activity was expressed as U/g FW, where *U* = 0.01 OD_333_ min^–1^.

The activity of cinnamyl alcohol dehydrogenase (CAD) was determined using a test kit from Suzhou Keming Biotechnology Co., Ltd., Suzhou, China. Extract solution (1 ml) was added to 0.5 g of frozen powder, then centrifuged at 8,000 × *g* at 4°C for 10 min. Other reagents were added as instructed, and OD_340_ was measured as first OD, and second OD was measured after 5 min. CAD activity was expressed as nmol/min/g FW.

Cinnamoyl-CoA reductase (CCR) activity was determined using a test kit from Enzyme-Linked Immunosorbent Assay (ELISA) Co., Ltd., Suzhou, China. One milliliter of 10 mM pH 7.4 was added to 0.5 g of frozen powder, then centrifuged at 8,000 × *g* at 4°C for 15 min. The collected supernatant was the crude enzyme. Other reagents were added as instructed by the kit, and OD_450_ was measured. CCR activity was expressed as U/g FW, where *U* = 0.01 OD_333_ min^–1^.

The PPO and peroxidase (POD) activities were scientifically assayed according to the Venisse et al.’s (2002) method. An acetic acid–sodium acetate buffer (pH 5.5, containing 1 mmol/L polyethylene glycol, 1% Triton X-100, and 4% polyvinylpyrrolidone) was used as an extraction reagent. An amount of 5-ml extraction buffer was added to 2 g of frozen powder and kept for 30 min, centrifuged at 8,000 × *g* for 30 min at 4°C, and the collected supernatant was the enzyme solution. Then, 3 ml of 50 mmol/L acetic acid–sodium acetate buffer (pH 5.5) and 1 ml of 50 mmol/L catechol were added to 0.1 ml of the crude enzyme, and absorbance was immediately determined. The PPO activity was expressed as U/g FW, *U* = 0.01 OD_420_ min^–1^. To determine POD, 3 ml of 25 mmol/L guaiacol was added to 1 ml of a crude enzyme, after which 0.2 ml of 0.5 mol/L H_2_O_2_ was added, and the absorbance was determined. The POD activity was expressed as U/g FW, *U* = 0.01 OD_470_ min^–1^.

### High-Performance Liquid Chromatography Analysis of Phenolic Acids and Monolignin Contents

The phenolic acids and monolignin contents were scientifically determined using the Zhao et al.’s (2006) method with slight modification. Three milliliters of 70% methanol was added to 3 g of sample tissues and undergoes ultrasonication for 30 min at 40 kHz before being centrifuged at 10,000 × *g* at 4°C for 20 min. The resultant supernatant was collected and concentrated under nitrogen before being diluted with 70% methanol to 1 ml. The resulting 1-ml solution was collected into an automatic injection (2 ml) bottle by filtering using an organic membrane medium of 0.45 μm. High-performance liquid chromatography was performed using Waters Symmetry C18 (4.6 × 250 mm, 5 μm) column. Methanol (A) and 1% acetic acid (B) mobile phase condition was as follows: gradient elution at 0–10 min, 30% A, 70% B; 10–12 min, 45% A, 55% B; 12–15 min, 65% A, 35% B; 15–18 min, 30% A, 70% B; and 18–20 min, 30% A, 70% B with a flow rate of 1 ml min^–1^ and injection volume of 10 μl, controlled at 25°C. Cinnamic acid, caffeic acid, ferulic acid, sinapic acid, and *p*-coumaric acid were detected at 276, 325, 322, 325, and 310 nm, respectively, and *p*-coumaryl alcohol, coniferyl alcohol, and sinapyl alcohol at 273, 263, and 273 nm, respectively. A standard curve of a standard sample under the same condition was set, and the concentration of all phenolic compounds was determined in comparison with the standard curve. The amount of substance (μg) to cause a change in absorbance per minute as compared with the standard sample was referred to as the individual contents. The various phenolic acids and monolignin contents were expressed as μg/g FW.

### Determination of Total Phenol, Flavonoids, and Lignin Content

Scalbert et al.’s (1989) method was used to determine total phenols and flavonoid contents. Three grams of frozen tissues was carefully homogenized with a 5-ml solution of 0.5% acetic acid (CHCOOH) and 70% acetone (C_3_H_6_O) and then kept at 4°C for 24 h in the dark. The mixture was then centrifuged at 8,000 × *g* for 20 min at 4°C. The resulting supernatant was used for the determination of total phenol and flavonoid contents.

Total phenol was determined by adding 2 ml of 0.1% (v/v) Folin–Ciocalteu’s phenol chemical to 1-ml supernatant, then a volume of 2 ml of 7.5% Na_2_CO_3_ was added and heated for 5 min at 50°C. The absorbance, OD_760_, was measured. To estimate total phenol content, a gallic acid standard curve was used, and the result was expressed as milligrams of gallic acid equivalents 100 g^–1^ FW. To determine flavonoid content, 0.25 ml of AlCl_3_•6H_2_O (10%) and 0.15 ml of NaNO_2_ (5%) solution was mixed with a 3.5-ml crude enzyme. After 5 min, 1 ml of 1-M NaOH was also added. Absorbance was measured at 510 nm. A standard rutin curve was used to estimate the content of flavonoid and collectively expressed as milligrams of rutin equivalents 100 g^–1^ FW.

The method described by Hammerschmidt (1984) was used in this study to determine the lignin content. Three milliliters of 95% precooled was added to 1 g of the frozen powder, mixed very well, and centrifuged at 8,000 × *g* for 30 min at 4°C; the resulting precipitate was immediately collected (three times). After that, n-hexane–ethanol = 2:1 (v/v) was added to precipitate and again centrifuged three times continuously. The final precipitate collected was dried in an oven for 48 h, then 1 ml of 25% bromoacetic acid glacial acetic acid solution was added to the dried samples and held in a hot water bath (70°C) for 30 min before stopping the reaction with 1-ml NaOH (2 mol L^–1^). Right after the addition of 2-ml glacial acetic acid, 0.1-ml hydroxylamine hydrochloride (7.5 mol/L) was added and immediately centrifuged. Finally, 0.5-ml supernatant was topped up with glacial acetic acid to 5 ml. The absorbance was immediately measured. The lignin content was expressed as OD_280_ g^–1^ FW.

### Statistical Analysis

All the determinations mentioned earlier were repeated three times. Averages and standard errors (±SE) of data were calculated using Excel 2010. Significance differences were analyzed by analysis of variance (Duncan’s multiple range test) using SPSS 20 (SPSS Inc., United States) (*p* < 0.05).

## Results

### Chitosan Treatment Reduced Weight Loss and Disease Index of Fruits During Healing

**FIGURE 1:**
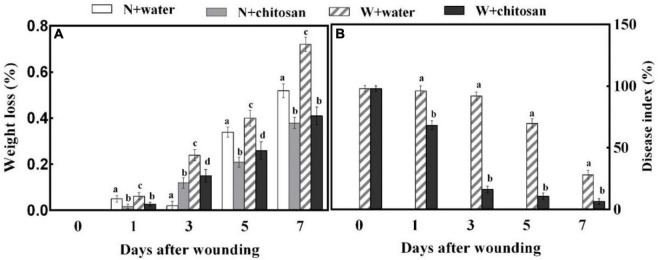
Effect of chitosan treatment on weight loss **(A)** and disease index of inoculated fruit with *P. expansum* **(B)**. Non-wounded and treated with sterile water (N + water), non-wounded and treated with chitosan (N + chitosan), wounded and treated with sterile water (W + water), and wounded and treated with chitosan (W + chitosan). Vertical bars indicate standard error of means. Alphabets indicate significant differences (*p* < 0.05).

Weight loss and disease index are crucial for evaluating the effect of wound healing on fruits. In this study, the weight loss for both chitosan treated and control fruits increased continuously during wound healing. On day 7, the weight loss of wounded chitosan-treated fruits was lower by 43% when compared with that of the control, but there was no significant difference compared with the non-wounded control (Figure 1A). The disease index of all fruits decreases as healing time increases, but the chitosan-treated fruits had a significantly lower disease index than the control fruits, which was 84.8% lower than the control at day 5 (Figure 1B). This is a clear indication that chitosan treatment promotes wound healing of apple fruits.

### Effect of Chitosan Treatment on Hydrogen Peroxide Content and Peroxidase Activity

**FIGURE 2:**
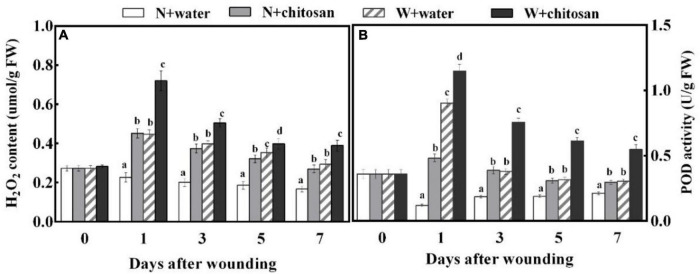
Effect of chitosan treatment on H_2_O_2_ content **(A)** and POD activity **(B)**. Non-wounded and treated with sterile water (N + water), non-wounded and treated with chitosan (N + chitosan), wounded and treated with sterile water (W + water), and wounded and treated with chitosan (W + chitosan). Vertical bars indicate standard error of means. Alphabets indicate significant differences (*p* < 0.05).

Hydrogen peroxide is an important signal molecule that induces a defense response. The H_2_O_2_ contents and POD activity of wounded fruits increased sharply on day 1 and declined gradually in all fruits, but the chitosan-treated fruits exhibited significantly higher values throughout the healing period. The H_2_O_2_ content on day 1 was 61% higher than the control, and POD activity was 94% more than the control on day 5, with a significant difference compared with their non-wounded fruits (Figure 2). These results show that chitosan treatment increases H_2_O_2_ content and POD activity at fruit wounds.

### Chitosan Treatment Increased Phenylalanine Ammonia-Lyase, Cinnamic Acid 4-Hydroxylase, 4-Coumaryl Coenzyme A Ligase, Cinnamoyl-CoA Reductase, and Cinnamyl Alcohol Dehydrogenase Activities

**FIGURE 3:**
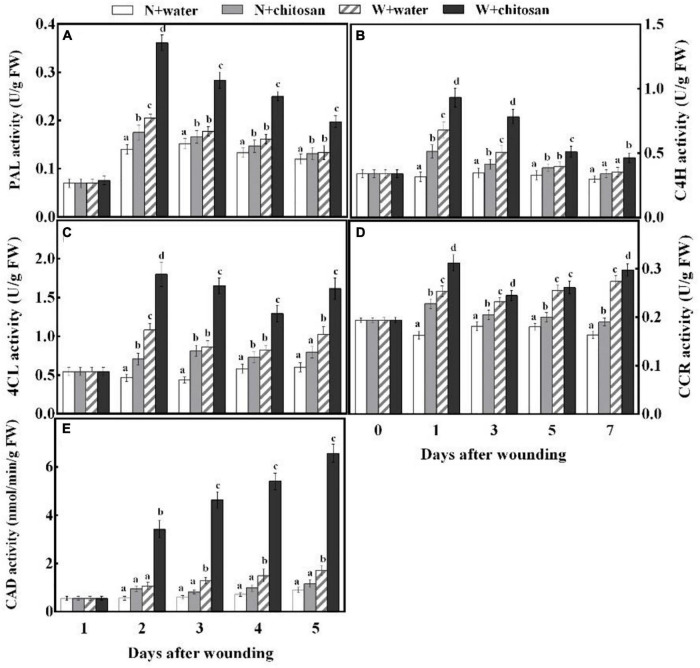
Effect of chitosan treatment on activities of PAL **(A)**, C4H **(B)**, 4CL **(C)**, CCR **(D)**, and CAD **(E)**. Non-wounded and treated with sterile water (N + water), non-wounded and treated with chitosan (N + chitosan), wounded and treated with sterile water (W + water), and wounded and treated with chitosan (W + chitosan). Vertical bars indicate standard error of means. Alphabets indicate significant differences (*p* < 0.05).

Phenylalanine ammonia-lyase, C4H, 4CL, CCR, and CAD are important enzymes in the phenylpropanoid metabolism to aid in the production of phenolic and lignin. PAL and C4H activities peaked on day 1 and declined gradually from days 3 to 7 for both chitosan-treated and control wounded fruits, with the chitosan-treated fruits recording significantly higher activities throughout the healing period (Figures 3A,B), which were 76 and 56.3% higher than the control at days 1 and 3, respectively (Figures 3A,B). There were significant differences compared with their non-wounded fruits on days 1 and 3, respectively. 4CL activity increased sharply at the initial stage, declined on days 3 and 5, and finally increased on day 7 for all fruits, but the chitosan-treated fruits showed significantly higher activity, that is, 91% higher than the control at day 3, with a significant difference compared with their non-wounded fruits (Figure 3C). CCR activity increased sharply at the initial stage, declined on the third day, and increased gradually from days 5 to 7 for all wounded fruits, but the chitosan-treated fruits showed significantly higher activity, which was 23% higher than the control at day 1; however, there was a significant difference in comparison with its non-wounded fruits (Figure 3D). The CAD activity of both treated and control wounded fruits increased continuously throughout the healing period; however, the chitosan-treated fruits recorded significantly higher CAD activities than the control (Figure 3E). Moreover, there was a significant difference in comparison with its non-wounded fruits. The highest value of the treated fruits was recorded on the seventh day, which was 2.8-fold higher than the control (Figure 3E). The results earlier show that chitosan treatment effectively elicited the phenylpropanoid pathway by increasing the activities of PAL, C4H, 4CL, CCR, and CAD at the fruits’ wounds during healing.

### Chitosan Treatments Elevated Phenolic Acids, Total Phenol, and Flavonoid Contents

**FIGURE 4:**
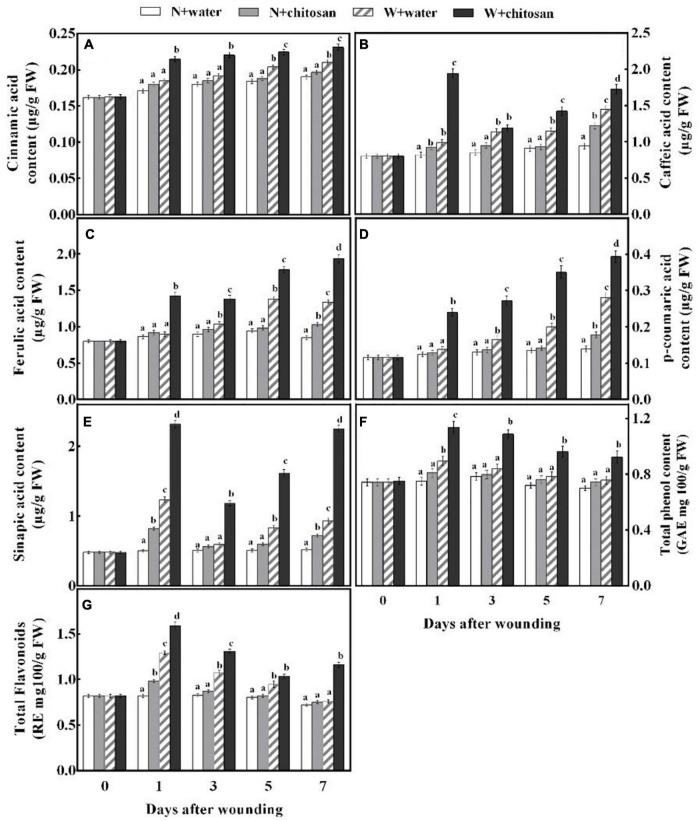
Effect of chitosan treatment on contents of cinnamic acid **(A)**, caffeic acid **(B)**, ferulic acid **(C)**, *p*-coumaric acid **(D)**, sinapic acid **(E)**, total phenols **(F)**, and total flavonoids **(G)**. Non-wounded and treated with sterile water (N + water), non-wounded and treated with chitosan (N + chitosan), wounded and treated with sterile water (W + water), and wounded and treated with chitosan (W + chitosan). Vertical bars indicate standard error of means. Alphabets indicate significant differences (*p* < 0.05).

Phenolic acids are essential substrates for healing tissues formation, which also have antifungal and antioxidant properties. Cinnamic acid, ferulic acid, and *p*-coumaric acid contents of wounded chitosan-treated and control fruits increase as the healing time increases, but the chitosan-treated fruits showed significantly higher acid contents, which were 16.2, 58.3, and 74.1% more than the control on day 7, respectively. Moreover, there was a significant difference in comparison with their non-wounded fruits (Figures 4A,C,D). Caffeic acid and sinapic acid content increased sharply on the first day, declined on day 3, and increased gradually afterward for all fruits, but the chitosan-treated fruits recorded significantly higher acid contents compared with the control fruit. Caffeic acid was 96.3% higher than the control on day 1, and sinapic acid was 1.4 fold more than the control on day 7, with significant differences compared with their non-wounded fruits, respectively (Figures 4B,E). The total phenol content of all wounded fruits peaked on day 1 and declined gradually from days 3 to 7 (Figure 4F). However, the chitosan-treated fruits recorded higher contents than the controls. The total phenol content of chitosan-treated fruit was significantly high when compared with the control by 30% at day 3. Flavonoid content of wounded fruits increased sharply on day 1 and decreased gradually, but the treated fruits increased slightly on day 7 (Figure 4G). Flavonoid content of chitosan-treated fruit was significantly higher throughout the healing time, which was 53% more compared with the control at day 7, with a significant difference compared with its non-wounded fruits (Figure 4G). The result indicates that chitosan treatment elevates the synthesis of five phenolic acids, total phenol, and flavonoid at the fruits’ wounds during healing.

### Chitosan Treatments Enhanced the Synthesis of Monolignins and Lignin

**FIGURE 5:**
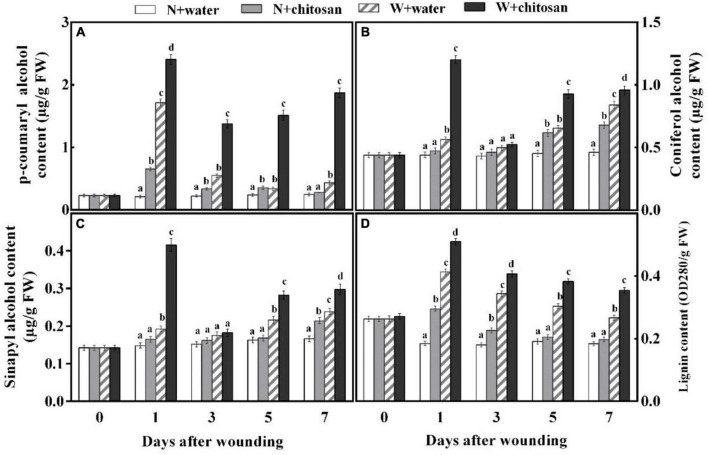
Effect of chitosan treatment on contents of *p*-coumaryl alcohol **(A)**, coniferyl alcohol **(B)**, sinapyl alcohol **(C)**, and lignin **(D)**. Non-wounded and treated with sterile water (N + water), non-wounded and treated with chitosan (N + chitosan), wounded and treated with sterile water (W + water), and wounded and treated with chitosan (W + chitosan). Vertical bars indicate standard error of means. Alphabets indicate significant differences (*p* < 0.05).

Coumaryl alcohol, coniferyl alcohol, and sinapyl alcohol are important monomers for the synthesis of lignin to increase the strength of cell walls. In this study, the contents of *p*-coumaryl alcohol, coniferyl alcohol, and sinapyl alcohol increased sharply on the first day, declined on day 3, and increased gradually afterward for both the treated and control fruits, but the chitosan-treated fruits recorded significantly higher contents compared with the control fruit (Figures 5A–C). *p*-Coumaryl alcohol content was 4.54-fold higher than the control fruits on day 3. On day 1, coniferyl and sinapyl alcohols were 1.13- and 1.56-fold higher than the control fruits, with significant differences compared to their non-wounded fruits, respectively (Figures 5A–C). The lignin content peaked on day 1 and declined gradually from days 3 to 7 for all fruits (Figure 5D). The chitosan-treated fruits recorded significantly higher content that was 33% more in comparison with the control at day 7, and when compared with its non-wounded fruits, there was a significant difference (Figure 5D). The result indicates that chitosan treatment promotes the synthesis of three lignin monomers and lignin of fruits during healing.

### Chitosan Treatment Accelerates Enzymatic Browning at the Wound Surface

**FIGURE 6:**
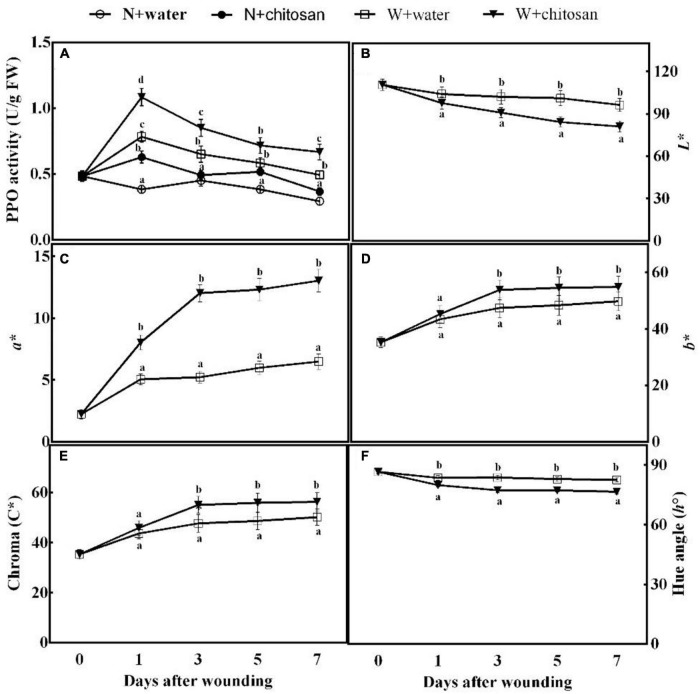
Effect of chitosan treatment on PPO activity **(A)** and L* **(B)**, a* **(C)**, b* **(D)**, Chroma C* **(E)**, and hue angle *h*° **(F)**. Non-wounded and treated with sterile water (N + water), non-wounded and treated with chitosan (N + chitosan), wounded and treated with sterile water (W + water), and wounded and treated with chitosan (W + chitosan). Vertical bars indicate standard error of means. Alphabets indicate significant differences (*p* < 0.05).

Browning at the wound surface is an indication of the oxidation of polyphenolic compounds. The activity of PPO peaked on day 1 and declined gradually from the third to the seventh day for all wounded fruits, but the chitosan-treated fruits showed significantly higher activity throughout the process of healing (Figure 6A). On day 7, chitosan-treated fruits had a significant increment of 35.1% compared with the control with significant difference compared with their non-wounded fruits (Figure 6A). Lightness (L*) and hue angle values of both the treated and control fruits decrease as time increases; however, chitosan-treated fruits were significantly low when compared with the control fruits (Figures 6B,F). Reddish–greenish (a*), yellowish–bluish (b*), and chroma values of all fruits increase as healing time increases (Figures 6C–E). The treated fruits showed significantly higher values than the control, which were 1.3-fold, 13.4%, and 15.5% higher than the control for reddish–greenish (a*), yellowish–bluish (b*), and Chroma, respectively, at day 3 (Figures 6C–E). The results indicate that chitosan treatment accelerates enzymatic browning at the wound surface.

## Discussion

In this study, wounding increased weight loss as a result of stress. However, wounded fruit treated with chitosan effectively reduced the weight loss during healing by forming a protective film on the wound surface (Figure 1). Our results are in line with those of Plainsirichai et al. (2014), who discovered that chitosan could form a thin protective film directly on the fruit wound surface, thereby entirely or partially blocking spores on the surface to limit water transpiration. Chitosan films are also effective gas barriers, which modifies fruit’s internal atmosphere by controlling the supply of O_2_, CO_2_, and moisture, thus reducing water transpiration and respiration and delaying ripening and senescence of fruits, which was evident in sweet cherries, strawberries, and pomegranates during storage (Pasquariello et al., 2015; Petriccione et al., 2015; Varasteh et al., 2017). In addition, lignin accumulation limits fruit water evaporation when formed on a wounded surface (Xue et al., 2019). Thus, the gradual accretion of lignin at the wound site of fruit in the present study also reduced water loss.

The results show that wounded fruit exhibited a high disease index compared with control. Chitosan-treated fruits had a significantly lower disease index than the control fruits (Figure 1B). Chitosan acts as a fungicidal treatment to destroy pathogens (Li et al., 2015). Chitosan can bind to plant defense proteins to induce phytoalexin and PR proteins: chitinase, β-1,3-glucanase, and lipoxygenase (Hidangmayum et al., 2019; Gu et al., 2021). For instance, chitinase, disease resistance protein, cellulose synthase-like protein E6, PPO, etc., were induced to stimulate the synthesis of PR proteins in grapes; however, these genes also stimulated H_2_O_2_, JA, SA, and ET biosynthesis to induce resistance and promote the formation of barrier tissues to prevent pathogen invasion (Peian et al., 2021). Lignin deposition at wounds by chitosan also contributes to the reduction of disease index because lignin is a defense polymer that restricts pathogen penetration or growth by depositing around the penetrating fungi (Xie et al., 2018). Therefore, it is suggested that chitosan reduced the disease index of inoculated fruits by acting as a fungicide, deposition of lignin, inducing phytoalexin and PR proteins production, and accumulation of phenol compounds at fruits’ wound.

Phenylpropanoid metabolism aids wound healing by providing substrates for the synthesis of phenolic and monolignin with antifungal effects (Yang et al., 2020). PAL is a key enzyme initiating this metabolism by the deamination of L-phenylalanine to *trans-*cinnamic acid (Wang et al., 2020). *Trans-*cinnamic acid formed is directly hydroxylated by C4H to *p*-coumaric acid, which yields caffeic acid with the aid of *p*-coumaroyl shikimate 3-hydroxylase, and then caffeic acid is further generated into ferulic acid under *O*-methyltransferases. Sinapic acid is generated by converting ferulic acid into 5-hydroxyferulic acid by ferulic acid-5-hydroxylase, then further hydrolyzed by 5-hydroxyferulic acid *O*-methyltransferase into sinapic acid (Yang et al., 2020). These phenolic acids are converted to phenolic acids coenzyme A by 4CL and then catalyzed by CCR to yield corresponding aldehydes. The resulting aldehydes are catalyzed by CAD to form monolignins (*p*-coumaryl alcohol, coniferyl alcohol, and sinapyl alcohol) (Li et al., 2021). These phenolic acids and monolignin are precursors for suberin polyphenolic and lignin to strengthen cell walls. Coumaryl-CoA is also the precursor for the biosynthesis of flavonoids, which branch off from the phenylpropanoid pathway under the activities of chalcone synthase and stilbene synthase (Waki et al., 2020). In the present study, wounded fruits increased the activities of PAL, C4H, 4CL, CCR, and CAD (Figure 3). However, wounded fruits treated with chitosan had higher PAL, C4H, 4CL, CCR, and CAD activities as well as cinnamic acid, *p*-coumaric acid, caffeic acid, ferulic acid, sinapic acid, flavonoids, and total phenol contents compared with the control, respectively (Figures 3, 4). The current results agree with the work done by Li et al. (2021), where chitosan preharvest spraying increased the synthesis of cinnamic acid, *p*-coumaric acid, caffeic acid, ferulic acid, total phenol, and flavonoid contents at the wound site of harvested muskmelon by eliciting PAL and C4H activities. Chitosan is an elicitor that induces oxygen burst for the synthesis of H_2_O_2_ at the early stage of stress; however, the H_2_O_2_ induces PAL activity (Fooladi vanda et al., 2019). According to Lin et al. (2005), chitosan treatment increases the biosynthesis of signal molecules such as H_2_O_2_, JA, and SA by inducing the octadecanoid pathway to elicit PAL activity in rice cells. Furthermore, chitosan stimulates defense at the transcriptional level by inducing *VvLOX*, *VvCOI1*, *VvAOC*, and *VvAOS* genes to increase the synthesis of H_2_O_2_, JA, SA, ET, and abscisic acid in grapes and strawberries to promote the synthesis of phenolic compounds (Peian et al., 2021). Flavonoids and some phenolic acids have a direct effect on pathogens by acting as phytoalexins to restrict mycelium and spore germination (Zhang et al., 2020). Lignin is considered an essential healing structure to strengthen cell walls in the scope of fruit wound healing (Xie et al., 2018). Monolignins (*p*-coumaryl alcohol, coniferyl alcohol, and sinapyl alcohol) are polymerized into *p*-hydroxyphenyl lignin, guaiacol lignin, and syringyl lignin linked by ether bonds into a structure known as polymer lignin, which is strong enough to prevent pathogen invasion (Yang et al., 2020; Zhu et al., 2021). Moreover, lignin accumulation is known to limit water evaporation and restrict pathogen infections (Xue et al., 2019). Our results show that chitosan treatment enhanced the synthesis of *p*-coumaryl alcohol, coniferyl alcohol, and sinapyl alcohol, which accumulated into lignin (Figure 5). Similarly, Zhao et al. (2018) reported that chitosan stimulated the accumulation of lignin at the fruit wound site. POD is involved in the polymerization of cinnamyl alcohols to form SPP and lignin *via* the POD-mediated free radical coupling process during lignification in the presence of H_2_O_2_ (Yang et al., 2020). Therefore, high H_2_O_2_ content by chitosan in this study not only induces PAL but also activates POD in the oxidative cross-linking between substrates for lignification. The results of our study agree with that of Zhao et al. (2018) and Li et al. (2021), who reported that chitosan elicits enzymes to promote healing of wounds by lignin accumulation in muskmelon and citrus. Thus, it is suggested that chitosan treatment may promote the phenylpropanoid pathway by inducing the synthesis of signal molecules pathways at the fruit’s wound site to enhance the activities of enzymes and the synthesis of phenol compounds and lignin.

Polyphenol oxidase catalyzes the oxidation of tyrosine to quinones, which have stronger defensive power (Yan et al., 2018). Wounded fruits increased PPO activity. Moreover, our results show that wounded fruits treated with chitosan further increased the activity of PPO (Figure 6A). The activity of PPO not only induces resistance but is also the main cause of enzymatic browning in the presence of phenolic compounds (Xu et al., 2020). The phenolic compounds produced by the phenylpropanoid pathway are further catalyzed by PPO to produce quinones, causing browning. Therefore, the high accumulation of phenols and higher PPO activity in this present study led to the production of more quinones and enzymatic browning. Enzymatic browning reactions lead to the formation of water-soluble brown, gray, and black color pigments. The a* and b* values increased, whereas the L* values decreased in this study (Figure 6), indicating a transition from white to black or brown. Similarly, Pilon et al. (2015) reported that chitosan activated browning in apples by the rapid activation of PPO. We, therefore, infer that chitosan first elicited the phenylpropanoid pathway to produce more phenol compounds and enhanced PPO activity to stimulate the oxidation of phenols to quinones, causing color change (brown pigmentation) at the wound surface of fruits. Moreover, the quinones have a defense reaction against fungi (Xu et al., 2020), that can contribute to the reduction in disease (Figure 6B).

## Conclusion

Chitosan treatment significantly elicited the activities of PAL, C4H, 4CL, CCR, and CAD and promoted the synthesis of cinnamic acid, ferulic acid, caffeic acid, *p*-coumaric acid, sinapic acid, *p*-coumaryl alcohol, coniferyl alcohol, and sinapyl alcohol, as well as total phenols, flavonoids, and lignin at the wounds of apples. Again, chitosan activated PPO activity, which stimulated enzymatic browning at fruit wounds. Moreover, the accumulation of phenolics and lignin polymer at wound surface prevented excessive water loss fruits and reduced disease index of *P. expansum* inoculated fruits. The findings in this study will add up knowledge in the scope of fruit wound healing and effective agricultural use of chitosan, considering its safe, edible, biodegradable, and non-toxic.

## Data Availability Statement

The raw data supporting the conclusions of this article will be made available by the authors, without undue reservation.

## Author Contributions

SA, SX, and RO designed the experimental setup, performed the experiments and analyzed the data. SA wrote the major part of the article. YB designed the experiments, conceived the idea, and revised the article. YB, FK-A, YZ, and DP conceived the idea and revised the manuscript. All authors have read and agreed to the published version of the manuscript.

## Conflict of Interest

The authors declare that the research was conducted in the absence of any commercial or financial relationships that could be construed as a potential conflict of interest.

## Publisher’s Note

All claims expressed in this article are solely those of the authors and do not necessarily represent those of their affiliated organizations, or those of the publisher, the editors and the reviewers. Any product that may be evaluated in this article, or claim that may be made by its manufacturer, is not guaranteed or endorsed by the publisher.
